# Authors’ reply for “Considerations about causality in observational studies”

**DOI:** 10.1016/j.lana.2021.100137

**Published:** 2021-12-08

**Authors:** Bo Wen, Rongbin Xu, Yao Wu, Micheline de Sousa Zanotti Stagliorio Coêlho, Paulo Hilario Nascimento Saldiva, Yuming Guo, Shanshan Li

**Affiliations:** a School of Public Health and Preventive Medicine, Monash University, Level 2, 553 St Kilda Road, Melbourne, Victoria 3004, Australia; b Department of Pathology, Faculty of Medicine, University of São Paulo, São Paulo, Brazil

We thank Bruna Venturin for the comments regarding our work published recently.^1^ The letter enables us to clarify some of the results and issues in more detail.

Generally, Venturin's Correspondence focused on the study design and the causality of the present work. First, we admit that this is an ecological study and we obtained daily time-series data to estimate the association. However, it would be more accurate and necessary to describe the study design as “case-crossover”, or “time-stratified case-crossover” as we have been stated in the method part. This method has become one of the most common study designs for environmental epidemiological studies.^2^, ^3^, ^4^ The study design was different from the traditional time-series design used in ecological studies as the self-matched design was applied. Thus, it could be misleading to use “ecological study”. Secondly, consistent with extensive studies focusing on the health impact of environmental exposure, we used “association” thoroughly instead of “effect” so as not to mislead readers that a causal effect of temperature on hospital admission for renal diseases was estimated. Similarly, we used “assuming a causal relationship” to describe the results of “attributable fraction” to prevent misunderstanding about the causation.

As for the temporality, Venturin's Correspondence emphasized that the exposure was not prior to hospitalizations and may not trigger renal diseases immediately. However, daily mean temperatures of seven days prior to the hospitalization day were used as the exposure in our study and the average level of exposure on the case periods was compared with control periods. Thus, temporal ambiguity would be difficult to occur. Moreover, although biological mechanisms for the association between ambient temperature and hospitalization for renal diseases were not clear, it should be noted that hospitalization for renal diseases would occur when the increase of temperature triggered acute kidney diseases and exacerbated symptoms or stages of patients with underlying renal diseases. Nevertheless, further research is still in need to deeply understand their association.

Many other issues have been discussed by the Correspondence. The percentage change in risk of hospitalization for renal diseases was calculated as (RR−1)×100% in the present study, where RR is the relative risk. Therefore, our calculation is correct with (1.009–1) × 100% = 0.9%. More importantly, we would like to use the attributable fraction to describe the health burden associated with exposure, because it is easier to understand by policy makers and general readers.^5^ It was found that over 7% of hospitalizations could be attributable to temperature when assuming a causal relationship. As a result, the implementation of relevant public health policies is of great importance in the context of climate change. We agreed that there may be a difference between males and females regarding willingness to seek medical care. We also understand that the Brazilian Unified Health System (BUHS) has a hybrid as well as complicated structure, which may distribute unequally among different administrative areas. However, our results are not likely to be affected by these differences as we used a self-matched design within the same city.^6^ Nevertheless, as we have stated in the limitations, the present study only covered 80% of the Brazilian population, bringing a potential selection bias.

Finally, we would like to thank Bruna Venturin for the comments and we agree that more studies and analyses are in need, especially in low- and middle-income countries like Brazil, to provide evidence for the implementation of public health policies.

## Declaration of Interests

We declare no competing interests.
